# Persistence of touch deposits on a range of substrates assessed using an optimised application of Diamond Dye™

**DOI:** 10.1007/s00414-026-03871-5

**Published:** 2026-06-18

**Authors:** Heidi Monkman, Roland A.H. van Oorschot, Dion Latte, Mariya Goray

**Affiliations:** 1 College of Science and Engineering, Flinders University, Sturt Road, GPO Box 2100, Bedford Park, Adelaide, South Australia, SA 5042, 5000 Australia; 2 Office of the Chief Forensic Scientist, Victoria Police Forensic Services Department, Macleod, VIC Australia; 3 School of Agriculture, Biomedicine and Environment, La Trobe University, Bundoora, VIC Australia

**Keywords:** Diamond Dye™, Cell persistence, Method optimisation, Substrate impacts, Duration impacts

## Abstract

The persistence of biological material on various substrates over extended periods of time and the visualization of cellular degradation are an important area of consideration in DNA-TPPR research. This study investigates long-term cell persistence of touch deposits and the ability to visualise them on different substrates. A key focus of the study was optimisation of the Diamond Nucleic Acid Dye™ (DD) application to minimise disruption to cells while ensuring effective cell visualisation. Three spray methods were tested across seven distances, with performance evaluated based on fluorescence intensity and the spread of cellular material outside of the deposit circle.

Using the optimised method persistence of touch cells on six substrates: glass, plastic, melamine, aluminium, leather, and cotton were assessed at several timepoints up to a year including effect of respraying.

Results revealed diminishing and significant variation in. cell persistence across substrates, with cotton and leather displaying lowest persistence. Further, the results of the re-spraying, point to the need for respraying items after initial staining to ensure the best visualisation of cellular material over time. The DNA amounts in the deposits were then assessed, showing that the amount of DNA recovered was considerably less than what would be expected based on the cell counts.

This research provides valuable baseline data for forensic caseworkers to prioritise, where possible, sample collection based on substrate-specific cell persistence. Additionally, the study aids the work towards establishing best practices for Diamond Dye™ application to maximise visualisation efficiency with minimal cell disruption.

**Supplementary Information:**

The online version contains supplementary material available at 10.1007/s00414-026-03871-5.

## Introduction

The persistence of cells and DNA on surfaces is influenced by many different factors, including substrate types and their physicochemical properties [1–5]. In a forensic context, the topography of a substrate, its roughness, wettability and chemical surface interactions play roles in the deposition, adherence and persistence of biological material. Rougher surfaces with increased surface area and microscopic crevices tend to trap more cellular material, potentially enhancing cell and DNA preservation [6–8]. Conversely, highly hydrophobic surfaces may repel aqueous components of biological fluids, affecting the initial deposition of DNA-containing material [9]. Additionally, the strength of molecular interactions between the biological material and the surface can promote stronger adhesion [1, 10, 11]. Further, post-deposition, environmental conditions such as temperature and humidity and physical actions with the surface will either enhance DNA preservation or accelerate its degradation, or loss from, the substrate surface [8, 12–17]. The preservation of DNA over time is crucial in forensic investigations where a crime scene may not be processed for days and samples may be stored for months or years, depending on case circumstances, laboratory practices and priorities.

To maximise the chances of successful DNA recovery, it is not only important to understand DNA persistence over time but also to accurately localise and visualise the biological material on evidence items before sampling, to optimise DNA recovery. Recently, the use of Diamond Nucleic Acid Dye™ (DD) has been described as possible means of visualising touch deposits on items of evidence [18–21]. This fluorescent dye has also demonstrated utility in shedder status assessment [22]. Currently, there are limited studies investigating the use of DD on different substrates in light of possible persistence issues [23–26]. Generally, when DD is used, the stained cells are visualised and counted soon [19, 21] after exposure. During the course of conducting case work, there may be times when DD- exposed items will need to be re-examined sometime after the initial examination. It is currently unknown how long after the initial exposure of cells to DD they remain fluorescent and visible. Further, if the fluorescence intensity declines over time, is this dependent on the deposit substrate and if so, is it possible to effectively re-apply DD to visualise the cells.

This study aimed to investigate the most effective method of applying DD, and the persistence of touch cells on six different surfaces (glass, plastic, melamine, aluminium, leather and cotton) over a twelve-month period assessed using DD staining and imaging. Additionally, we aimed to explore DD fluorescence retention across the surfaces at 20 different time points over 6 months and a further 6 months after respraying the samples, including the assessment of the utility of DD respraying, at two different time points (6 and 12 months), thereby contributing to a more comprehensive understanding of the factors affecting persistence and fluorescence of biological material on these regularly encountered substrates.

## Materials and methods

### Diamond Dye™ spraying process optimisation

**Fig. 1:**
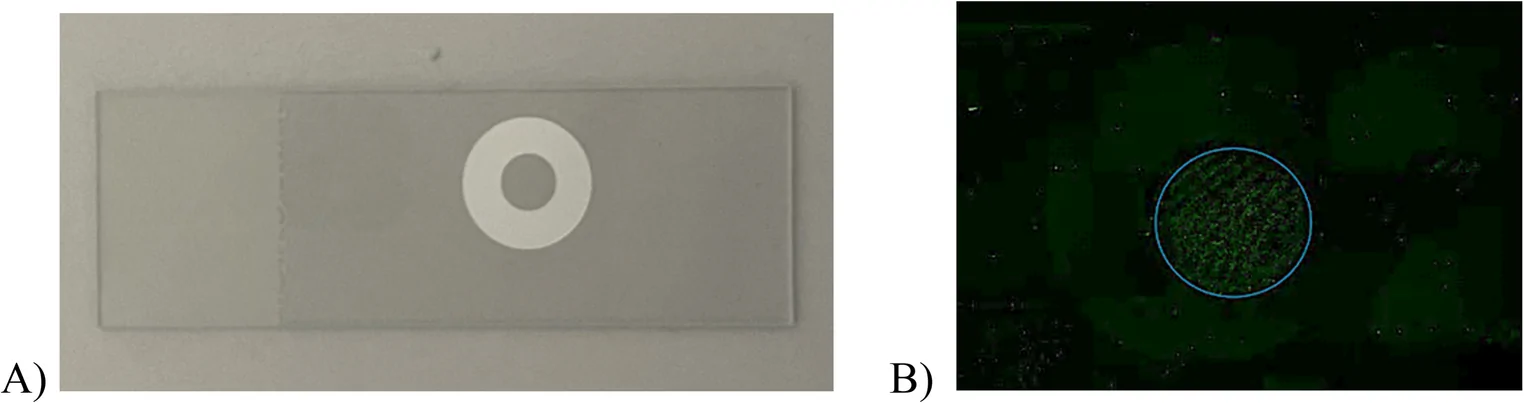
A slide with the 11 mm ring on the underside (**A**) and a representation of the cells counted outside of the circle that has been superimposed on the outline where the ring had been (whole slide not shown) (**B**)

In order to assess the movement of cells from their original deposition location during DD spraying, optimisation was undertaken prior to the persistence study. Glass slides (Livingstone, Australia) were cleaned with 1% hypochlorite, 70% ethanol and deionised water followed by exposure to the UV light for irradiation for 15 min [27]. Saliva was deposited in 5 µl amounts onto 63 glass slides inside the deposit area indicated with a 11 mm diameter hollow ring dispense-a-label sticky dot (Avery, Australia) that was attached to the underside of the slide (Fig. 1). The saliva was then left to dry for approximately 24 h.

The solution of Diamond Nucleic Acid Dye™ (Promega) was prepared in a 1 in 500 dilution of 10,000X stock solution in 70% ethanol and de-ionised water. Image collection was undertaken using DinoLIGHT (EDGE AM4115T-GFBW, AnMo Electronics Corporation) with an excitation of 480 nm and an emission filter of 510 nm at 50x magnification. Three different application methods were used to apply DD on glass slides: a small perfume bottle (single spray) sprayed once and twice, and a Voilamart HS08 mini air compressor (Voilamart™) that allows for continuous application sprayed twice over (in a back-and-forth motion) the surface for approximately 2 s (total time).

To determine the potential impact of the application distance from the target area on the visualisation of cells and/or their displacement from the deposit area, each of the three application methods were tested three times at each of the following spraying distances: 3 cm, 6 cm, 9 cm, 12 cm, 15 cm and 20 cm. Stained slides were air-dried for approximately 30 min and then imaged. The number of cells outside of the deposit circle (Fig. 1B) was then counted and compared across the different applications and distances to determine which application and distance combination produced the lowest movement of cellular material during DD application.

### Persistence of touch on different substrates

**Fig. 2:**
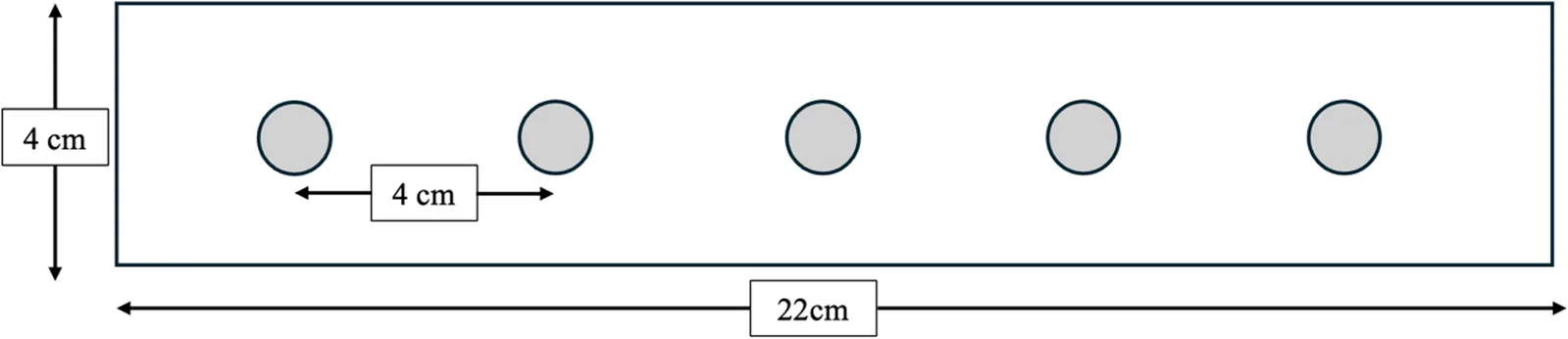
A visual description of the set up for the creation of five repeats of deposits on each of the substrates, excluding glass where individual slides were used per repeat

The persistence of touch cells was tested on six surfaces: glass slides, plastic transparency film (Nobo, Australia), aluminium (black), melamine (black), leather (textured) (black) and cotton (dark blue). All materials were of a dark colour where possible, with the exception of glass and plastic, which were imaged with the black microscope base as a background to aid cell visualisation. All surfaces were UV treated (BLX Bio-Link Crosslinker, Vilber) on both sides for 30 min period of time prior to cell deposition. For four of the five substrates (4 cm x 22 cm), excluding glass slides, the deposits were prepared by depositing a thumbprint of the dominant hand inside the five 11 mm hollow sticky rings that were attached on top of the substrate surface. The deposit rings were approximately 4 cm apart (Fig. 2). For the glass slides, an individual glass was used for each deposit.

Prior to deposition, a single participant washed their hands and waited 15 min. During the wait period, donors were instructed to not eat, drink, wear gloves or use hand sanitiser but could carry on their normal office activities. This process was repeated before each subsequent deposit.

The deposits were stained with DD using a continuous spray application (approx. 2 s) 20 cm away from the surface (determined from results of 3.1) utilising Voilamart air compressor.

Samples were accessed during each of the 20 tested time intervals over 12 months using ImageJ software (see Section "ImageJ Settings"). The tested timeframes post application of DD included immediate, 15 min, 30 min, 1 h, 2 h, 6 h, 24 h, 30 h, 48 h, 1 week, 2 weeks, 1 month, 2 months, 3 months, 4 months, 5 months, 6 months and 12 months. Additionally, at the 6 and 12 months’ time points, the DD was re-applied to each surface, and assessed after 30 min, to test if any apparent loss of cells and fluorescence is due to cell loss or reduction in fluorescence intensities. After 12 months of storage (and post 2nd re-spraying), the substrates were sampled using the wet-dry swabbing method for DNA quantification (see Section "Sample processing"). The latter was done to allow the comparison of DD cell abundance with retrieved DNA quantities. The samples were kept in cardboard boxes in a room that did not have windows and were not removed from this room at any time. The samples were taken out only during imaging (one at a time) and then immediately returned to the storage boxes until the next tested time frame.

### ImageJ settings

ImageJ is an image processing program developed by the National Institute of Health and the Laboratory for Optical and Computations Instrumentation (LOCI, University of Wisconsin) [28]. Settings were chosen based on Goray et al. [19] with circularity set at 0.6-1; however, each surface type had a different pixel threshold due to the different background fluorescence observed. The threshold ranges were as follows: glass (35–48), plastic (44–70), melamine (63–75), aluminium (30–46), cotton (79–90) and leather (60–65). After the threshold was applied the despeckle function was used to further eliminate any background fluorescence.

### Sample processing

DNA was collected from the deposits 12 months after deposition by wet and dry double swabbing of the surfaces using viscose swabs (Forensic Swab L, Sarstedt, Germany) that were combined for further analysis [29]. The wetting agent used was sterile water (LumaCina, Australia). DNA was extracted and quantified using PrepFiler™ (ThermoFisher) and Quantifiler Trio™ (Applied Biosystems), respectively, as per manufacturer recommendations. The total amount of DNA in the sample was calculated by multiplying the DNA concentration by the extract volume (50 µl).

### Data analysis

Deposited cell number changes, at different time points, were calculated by estimating average percentage decrease or increase at each time point for each of the substrates. For this purpose, initially the total number of cells deposited was estimated by counting all the stained cells either immediately or up to 24 h post staining. While for many of the deposits the total maximum cell number was observed immediately after staining, for several deposits DD stain incorporation into the cells took a longer period of time, with increases in cell numbers observed between immediate and 24-hour periods.

Thus, for all deposits and replicates, the time point at which the highest number of cells were observed (from immediate to 24 h depending on surface and repeat), after DD application, was designated as the maximum cell number and assumed to have been the total cell number for that replicate. This maximum cell number was then used for further analysis (the time point with the highest cell number used in the calculations can be found in Supplementary data 1; highlighted in purple). From this maximum cell number deposit, it was then calculated if the cell numbers were increasing or decreasing at preceding and succeeding time frames. For example, for glass surface and replicate 4 (Supplementary data 1), the maximum cell count of 40 cells was observed at 24-hour time post deposit. This deposit was, therefore, assigned as maximum cell number and cell numbers prior to this deposit were assigned percent of the total deposit indicative of cell visualisation increase, which in this instance was from immediate to 24 h (i.e., at immediate time 34 cells were visualised and assigned 85% of the maximum). Conversely, after the maximum cell deposit time frame, continued persistence was estimated (e.g., for glass repeat 4, 35 cells were observed at 30 h post deposit which is 88% of the maximum deposit of 40 cells that shows a 12% loss of cell visualisation). The increases and decreases in cell visualisation were calculated for each time point as percent of cells observed compared to the maximum cell number. For several repeats, cell number increases were seen up to 24 h (where maximum cell count was observed). To standardise the assessment, cell persistence estimation was initiated at 30 h post deposit for all replicates (Supplementary data 1). From this timeframe, average cell persistence per substate type was assessed. First the percent difference between cell count assessed and that repeats cell count maximum, as described above, was calculated (described as “Percentage of cells continued to be visualised” in the Supplementary data 1). Then, the average for the 5 repeats for each set of variables was estimated (described as “Average percentage of cells of cells continuing to be visualised” in the Supplementary data 1).

The normality of the data was tested using a Shapiro-Wilk test with a significance level of < 0.05 (SPSS v.29). An ANOVA was used to determine if there were any differences between the spray devices with a significance level of *p* ≤ 0.05. (SPSS v.29). The Friedman test was conducted to assess differences in persistence between substrates (χ²(df) = 1, *p* ≤ 0.05 (SPSS v.29).

## Results and discussion

### Spray device and application optimisation

The number of cells outside of the deposit circle (due to displacement during DD application) was estimated for each of the three spraying methods and the distances of application, by taking images and counting the number of cells outside of the deposit circle. Due to the spread, and in order to encompass all the cells displaced during DD application, the number of images taken per deposit ranged between 10 and 13 (*n* = 693).

The number of cells outside of the circle ranged between 9 and 140 (av. 51) for single spray with perfume bottle, between 16 and 76 (av. 41) for double spray with perfume bottle and 5 to 193 (av. 45) after the use of the continuous spray device (Table 1). Significantly more cells were found outside of the deposit areas when using single spray perfume device compared to the continuous device (*p* < 0.05). Fewer cells were found outside deposit after two sprays than continuous device application, however, these differences were not significant. The distance of the device to the deposit area was significant for all spray devices tested (*p* < 0.05). In general, as the distance from deposit to the device increased, the number of cells displaced outside the deposit decreased. The smallest cell displacement was observed with the continuous spray device and the greatest distance of 20 cm (Table 1).

**Table 1:** The number of cells detected outside the deposit circle for each distance and each spray device

Distance	Repeat	Perfume bottle Sprayed once	Perfume bottle Sprayed twice	Voilamart air compressor Sprayed continuously
3 cm	1	58	31	193
	2	140	51	107
	3	86	38	85
6 cm	1	55	36	75
	2	52	38	42
	3	58	41	45
9 cm	1	93	76	19
	2	51	51	31
	3	63	65	39
12 cm	1	27	34	36
	2	35	19	28
	3	43	48	27
15 cm	1	48	31	18
	2	32	49	16
	3	21	16	25
20 cm	1	35	20	5
	2	15	47	10
	3	9	41	12

The analysis of spray patterns and cell movements are required in consideration of the potential cell losses associated with each method and the impact it may have on trace DNA recovery. When minute amounts of DNA are present on a surface or an exhibit, collection maximisation is of importance and any losses should be prevented. Assessing the spraying distances, not surprisingly, at close proximity the force of the dye dispersion resulted in the greatest displacement of the cells, as most evident from the results of 3–12 cm distances (Table 1). Looking at the continuous spray device exclusively, significantly more (*p* ≤ 0.05) cellular displacement was observed at 3 cm distance compared to the other distances, likely due to the high working pressure of 15–50 PSI during device application [30]. The possibility of displacement of biological material on a substrate by air pressure has previously been reported [31]. As the distance between the test devices and the deposit surface increased past 12 cm, cell displacement decreased gradually with the smallest cell displacement noted for the Voilamart device (av. 14 cells for distances of 15–20 cm). Based on these results, the continuous spray device used at the distance of 20 cm represents the best method (of those tested) for cell staining on small target areas during item examination. This is especially to avoid: (a) loss of cells from small items, and (b) displacement of the cells from the deposit areas to areas and surfaces where they did not belong for larger items; resulting in loss of cells or greater sampling areas (and more background DNA collected). The latter point (b) is especially relevant to situations where the location of biological material may be of significance such as during of activity level considerations.

The observed data for single spray with perfume bottle revealed an inverse relationship between tested distance and cell fluorescence intensity. This was also noted, to a lesser degree, with the Voilamart device. As the distance increased, the fluorescence signal diminished, likely attributable to the broader dispersion of the dye over a larger area. This expanded dye distribution resulted in a lower concentration of dye per unit area, consequently reducing the overall fluorescence coverage and intensity detected from the cells. Nevertheless, the introduction of a second spray application markedly enhanced visualization; both with the two sprays of the perfume bottle and the continuous device. This improvement can be attributed to the deposition of an increased concentration of dye per unit area, which effectively increased the overall fluorescent signal and improved the clarity of the cellular imaging.

A notable issue observed with the perfume bottle methods was the inconsistent distribution of the dye. This irregularity manifested as large droplets and non-uniform spray patterns, resulting in uneven fluorescence intensities across the surface. In contrast, the Voilamart application method largely mitigated these problems, producing a more uniform dye distribution and, consequently, a more consistent fluorescence pattern. Our results concur with those of Young et al. [32], who previously compared 15 spray devices and demonstrated that a continuous spray device reduced negative effects such as large droplets and uneven and low intensity during visualising. Based on the results, an application of the dye with the continuous spray device at 20 cm distance is recommended, particularly when treating expansive surface areas. This approach ensures more optimal coverage and enhances the overall effectiveness of the fluorescent visualisation.

### Touch cell persistence on different surfaces over time

**Fig. 3:**
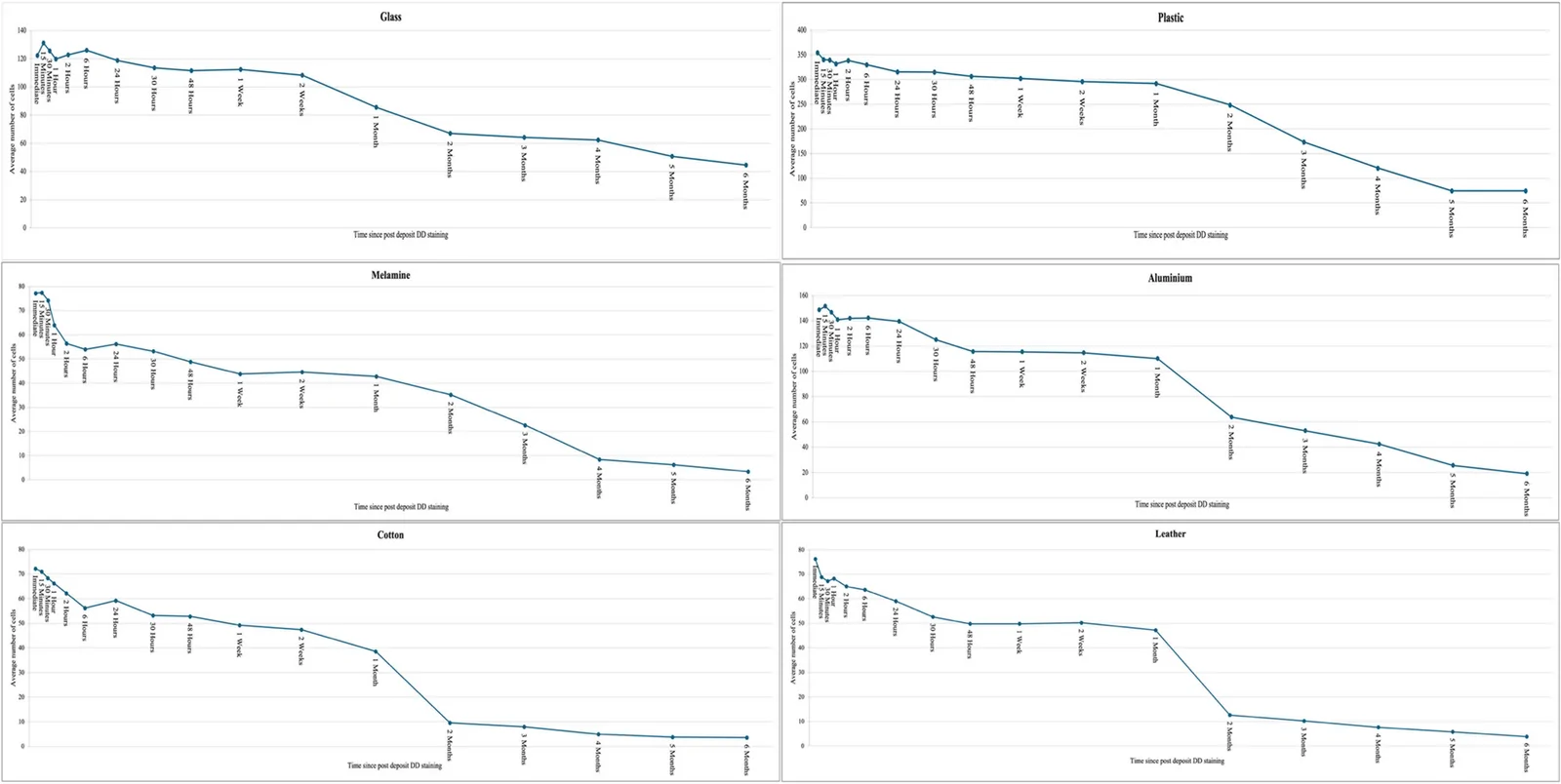
Percentage of visualised cells (average of 5 deposits) remaining at each time point until 6 months (before being resprayed) in hours

The ability to visualise stained cellular material on the six surfaces commonly encountered at crime scenes over a period of one year was investigated using the cell counts at each different time point (Fig. 3, Supplementary data 1). The persistence of the cells was also assessed during two re-spraying events at 6 and 12 months (Sect. 3.3). At initial time point, on average (across all 5 replicates), 122, 354, and 148 cells were detected on the flat, non-porous surfaces: glass, plastic and aluminium, respectively; and an average of 77, 76 and 72 cells were detected on the rough, porous surfaces: melamine, leather and cotton, respectively (Supplementary data 1 and 2). The textured/porous substrates, in general, had lower number of cells visualised compared to those that were flat and non-porous. This may be due to fewer cells being deposited as a consequence of a lesser area of contact (i.e., only contact with the raised surface areas and not the grooves) and/or because the cells settled into the grooves or lower layers of the substrates and were not accessible to DD staining.

Post-staining, there was an initial enhancement in visualisation up to 24 h that was substrate-specific (see Supplementary data 1 and 2). This gradual improvement in visualisation suggests a time-dependent process of dye penetration into the cells and subsequent DNA binding. The observed trend implies that the dye molecules slowly diffuse through cellular membranes and progressively associate with nuclear DNA, resulting in an incremental increase in fluorescent signal strength for these substances. These results suggest that if the precise number of cells on the surface is critical, for example, during shedder testing, it may be pertinent to delay cell counting to 30 min post-staining, depending on the substrate on which the deposit resides.

First losses in visualisation were noted, for some of the replicates, starting at 15 min post-staining (Fig. 3 and Supplementary data 1 and 2). After initial losses, for most of the substates, cell visualisation stabilised for a period of time ranging from few days to a month and then progressive decline in cell visualisation was noted until the re-spraying at six months’ time point. Prior to respraying, 5% to 32% of the initial deposits could still be visualised for the tested substates.

Overall, the greatest to lowest cell losses (at six months before respraying) were noted in the following order for the tested substrates: melamine, cotton, leather, aluminium, plastic and glass. For every substrate, cell visualisation declined significantly between initial time point and six months (*p* < 0.05). Further, while for some substrates these losses were gradual, for example, on plastic substrate 51% of cells were lost by the 3- months’ time point, for cotton 88% of the cells were lost. Observed trends in cell losses between substrate types may be influenced by factors such as surface topology, chemical interactions between cells and the substrate, and the presence of potential inhibitory or damaging compounds within the surface composition. For instance, when certain materials are subjected to contact forces exceeding approximately 20% of the substrate’s hardness, asperities may form, which can be either permanent or transient [33]. Such asperities can result in uneven surfaces and reduced contact areas for cell deposits as well as more shear- induced cell losses. The comparatively smooth surfaces of the glass, melamine, aluminium and plastic in this study may have thus allowed for better persistence once deposited. It should be noted that small fluctuations in cell counts between adjacent time points may also reflect inherent variability associated with image acquisition and cell counting.

### Re-application of Diamond Dye™ to enhance visualisation for targeted sampling

**Fig. 4:**
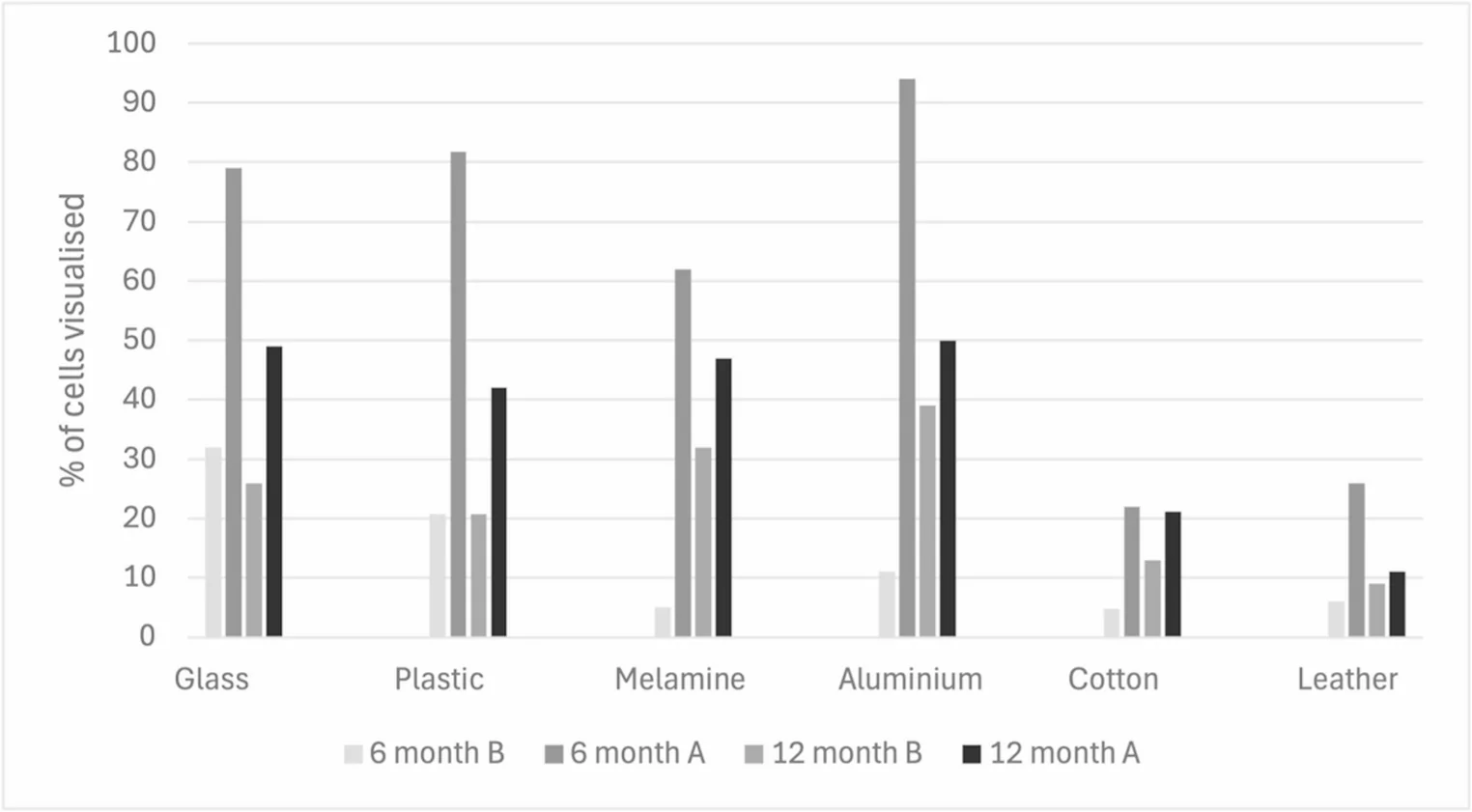
The effect of respraying the substrates on visualisation of cells at 6 months and 12 months estimated based on cell decreases compared to the initial deposits **B** Before respraying the substrates **A **After respraying the substrates

All deposits were visualised prior and post DD re-spraying at the two time points (6 and 12 months) across all substrates. The purpose of the respraying was to test if the losses noted during cell counts are associated with true cell loss or is an outcome of the diminished action of the initial application of DD. Only two timeframes were tested to limit potential impacts of reapplication of DD on the persistence of cells on a substrate. Averaging across all substrates (Supplementary data 1), re-spraying increased cell visualisation by 48% and 14% for the 6 and 12-month time frames respectively (Fig. 4; Supplementary data 2 for further substrate specific details).

At six months’ time point, respraying resulted in significant cell increases in visualisation of cellular material for all substates (*p* < 0.05). These increases were again observed for all substates at 12 months respraying, but was only significantly so for glass, plastic and leather (*p* < 0.05). These results suggests that cells experience fluorescence decay over time and are responsive to the reapplication of dye in cases where an item needs re-examination several months after the initial analysis and DD application. However, the respraying process itself may result in some cell losses that may need to be weighed against the need to visualise the cells that may have lost fluorescence at these later time points.

Overall, it appears that glass and leather resulted in the lowest cell persistence over the 12-month period compared to plastic, melamine, aluminium and cotton. Interestingly, glass and melamine had the smallest decrease in persistence from 6- to 12-months, indicating that for these surfaces most losses happen in the first 6 month. In contrast, for all other substrates major losses (of approximately half of the deposited cells) were observed between first respraying at 6 months and the second respraying at 12 months.

The persistence observed in the current study is similar to that of several other studies [8, 13, 14, 16, 26]. Lee et al. [14] measured persistence of blood and cultured human keratinocytes by counting alleles and using 7 different substrates in 3 different environments. While the findings of Lee et al. [14] cannot be strictly compared to the results here, due to the use of DNA profiling (rather than cell counts and quantification results) and the differences in biological materials investigated (blood and cultured keratinocytes rather than touch DNA), similar trends were noted. In their study Lee et al. [14] noted that in the indoor conditions partial drop out was observed by 8 weeks and by week 48 all substrates showed drop out and the larger losses were seen in synthetic leather, glass and plastic (compared to cotton/polyester, paper and aluminium). This is comparable to this study where leather showed lowest persistence at the six-month time point.

Similarly, Arsenault et al. [16] assessed persistence of trout cellular DNA, cell-free mouse DNA and mixture of both on various surfaces under differing conditions, including exposures to natural light, dark and high humidity environments. Sampling was conducted at 27 timepoints to track the decline in DNA quantities. Among the tested parameters, substrates stored in the dark most closely matched our study design. Again, the study comparisons are limited by differences in biological material (cellular trout DNA and cell free mouse DNA vs. human touch DNA) and processing techniques (trout DNA quantification vs. human cell counts and qualification). Notably on surfaces such as cotton, wood, carboard and paper, cell free DNA was undetectable after 6 months while on plastic, laminate and aluminium this DNA was still detectable at 12 months. In the same study, cellular DNA was detected on all substrates after 12 months. In contrast, in this study the highest amounts of DNA detected were with cotton and plastic substrates and the lowest with glass, leather and melamine (see Sect. 3.4 for more details). The possible discrepancies between the studies could be due to the use of trout and mouse cells and DNA compared to the human touch DNA utilised in this study.

### Cell counts and quantification results comparisons

The average DNA quantities recovered after 12 months from glass, plastic, melamine, aluminium, cotton and leather were 0.004 ng, 0.013 ng, 0.008 ng, 0.010 ng, 0.032 ng and 0.008 ng respectively (*n* = 5 per substrate; Supplementary data 1). When compared with the average cell counts for these substrates (av. of 60, 152, 36, 67, 16, and 8 cells, respectively), the recovered DNA quantities were markedly lower than expected, i.e.: approximately 99%, 99%, 96%, 98%, 67% and 83% less, assuming a typical cellular DNA content of 6 picogram per cell.

**Fig. 5:**
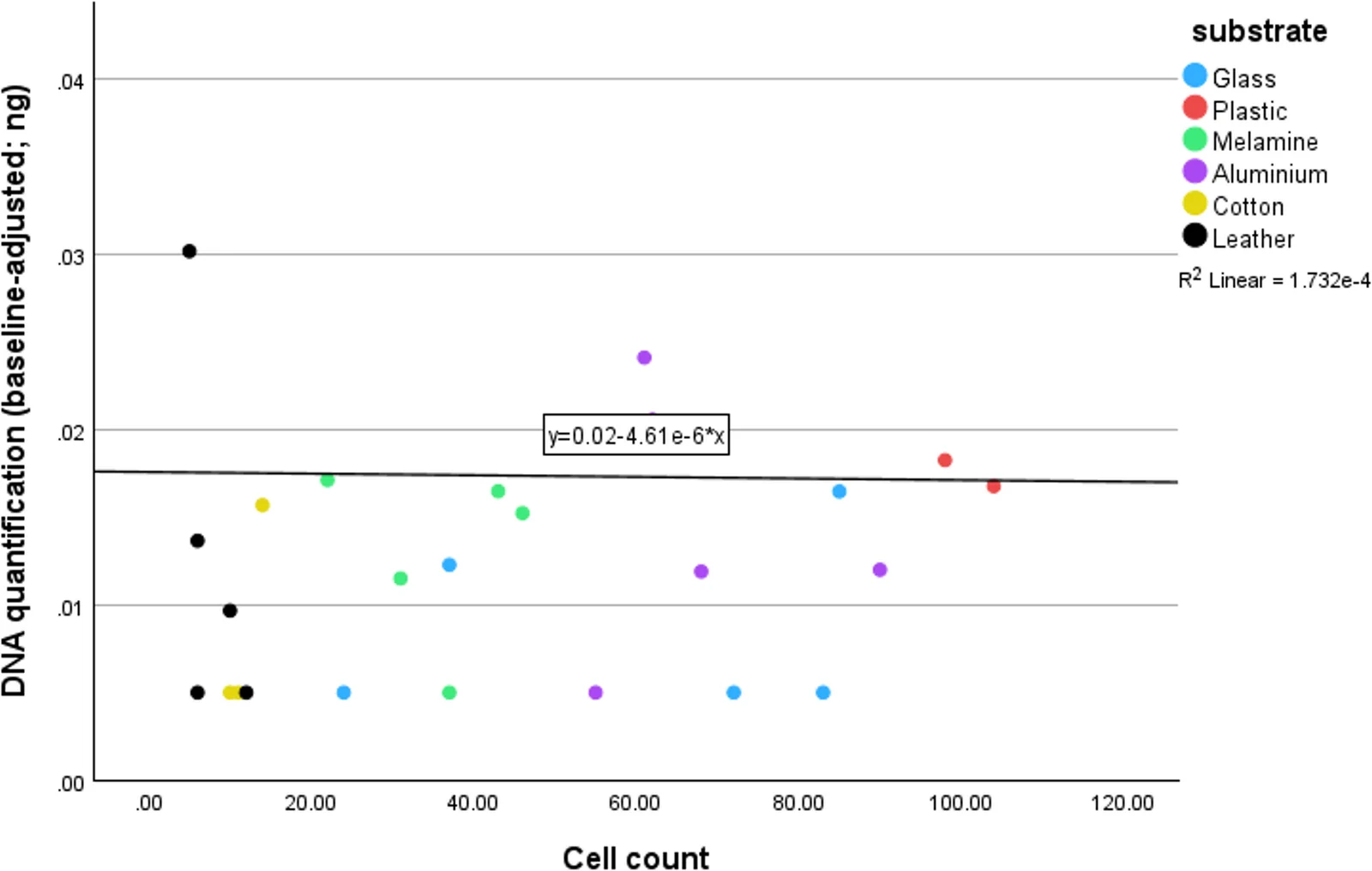
Scatter plot of cell counts against baseline-adjusted total DNA quantity (ng; +0.005 adjustment) for six substrates (and 5 replicates), with linear regression fit

To further investigate the relationship between cell counts and total DNA amounts recovered, linear regression was performed with DNA quantification yield baseline adjusted (0.005 ng was added to each DNA quantification value, ensuring that no result was zero and facilitating better visualisation and analysis). The resulting data were visualised in a scatter plot comparing cell count to DNA quantity for each substrate and replicate (Fig. 5).

Analysis found no significant association between cell count and DNA quantity, as evidenced by the virtually horizontal regression line and the minimal R^2^ value observed among different substrates and replicates. These results indicates that the variation in DNA yield is not explained by cell number, supporting the conclusion that DNA recovery efficiency is largely independent of the quantifiable cell count under these conditions. Based on cell counts, it can be hypothesised that DNA recovery should increase proportionally with cell number; as the number of recovered cells increases, the total amount of DNA is expected to rise accordingly. One possible explanation for the lack of correlation is DNA degradation in touch deposits. It is well established that DNA in touch samples is often degraded, which can substantially diminish quantification results leading to lower DNA yields than would be predicted from cell counts alone [34, 35]. Further, recovery and extraction efficiency and DD’s non-human specific DNA binding can also play a role [18, 21, 22, 35, 36].

## Conclusion

This study investigated the persistence of touch cells on six common surfaces over a period of one year while also assessing the utility of Diamond Dye™ cell visualisation method for this purpose, including method optimisation. We determined that the use of a continuous device at 20 cm provided the least disruption to the cells and the most consistent dye coverage. We found that the uptake and retention of fluorescence is dependent on the substrate type, with some surfaces providing a delay in the amount of cellular material visualised until up to 24 h after staining while others exhibited maximum fluorescence instantly. Additionally, each substrate had different times where there were large drops in fluorescence. Further, respraying the surface after a period of time appears to improve visualisation of cellular material with all substrates. The surface characteristics and their interactions with the deposited cells as well as the dye likely resulted in these discrepancies. To further understand substrate and dye interactions with touch deposits, future studies may benefit from inclusion of the effects of surface topography (roughness, porosity, shape and presence of gloves), chemical composition and functional group interactions as well as the hydrophobicity on both deposited cells and the dye that stains them. The persistence of cellular material visualised over time, even upon respraying showed a larger decrease with porous and textured substrates compared to non-porous substrates. Furthermore, future studies should also investigate the reasons for the lack of a relationship between the cell counts and quantification results and expand the analysis to include STR profiling and the relationship, or lack thereof, between cell counts and STR data.

Based on these findings when triaging exhibits for examination during the initial stages of the investigative process the vetting personnel should consider, not just the probative value of an exhibit, but also the potential losses associated with each item type. Thus, it may be that while the exhibit is low on the examination priority list and previously would have been stored until it was to be examined at a later date, now may be prioritised with the knowledge that if not examined soon, the potential evidence may be lost to future analysis.

## Supplementary Information

Supplementary Material 1.

Supplementary Material 2.
