# Supplementary material for: Persistence of touch deposits on a range of substrates assessed using an optimised application of Diamond Dye™
Source: Int J Legal Med. 2026 Jun 18;140(5):2709–18. doi: 10.1007/s00414-026-03871-5 (PMC13499732; doi:10.1007/s00414-026-03871-5)
Supplement: Supplementary file 1 [file 414_2026_3871_MOESM2_ESM.docx]

**Touch cell persistence over time specific to each of the different tested surfaces**

Two substrates (melamine and cotton) showed a small progressive enhancement in visualization for up to 30 minutes following the initial dye application. For the remaining surfaces, the maximum cell visualisation was achieved at 1, 2, 6 and 24 hours for leather, plastic, aluminium and glass respectively. However, majority of replicates for all substrates displayed maximum cell visualisation within 30 minutes (26/30 repeats) of DD application; highlighting that a minimum of 30 minutes post staining is required prior to analysis.

For the glass surface, the first loss in cell visualisation was noted at 15 minutes. Cell numbers remained stable from 30 hours and until 2 weeks post deposit. From week 2 onwards, there was a decline in visualisation of the cellular material at every time point until respraying occurred (section 3.3). Prior to respraying at 6 months’ time point, 32% of cellular material was still visualised on the surface.

For the plastic surface, in general, patterns similar to glass were observed, with first cell decreases noted at 15 minutes after deposit. The cell numbers remaining stable until 1-month time point were approximately 81% of the maximum cell number was still visible. From one month onwards, cell decreases were noted until respraying. Prior to respraying at the 6 months’ time frame, 21% of cellular material could still be visualised.

The melamine surface did not show any increase of fluorescence 30 minutes timepoint. The first cell losses were again observed at 15 minutes. For this surface, cell numbers remained stable (just over 60% of the maximum number on average) until 30 hours post deposit. From 30 hours onwards, an average loss of approximately 10% was observed at 2 weeks and continued monthly up to 5 months post deposit. At the six months’ time point (prior to respraying), only 4% of cellular material could still be visualised.

On aluminium, again, first losses were noted after 15 minutes. Interestingly, cell numbers remained stable at around 70% for up to one-month post-deposit, but at two months, a sharp decline occurred, with more than half of the cells no longer detectable. At the six months’ time point (prior to respraying), 11% of cellular material could still be visualised.

For both cotton and leather, once a decrease in cell number was observed at 15 minutes, these decreases continued at each successive time frame until 2 months at which time point, there was a sharp decline with over 37-41% cell loss. At the six months’ time point (prior to respraying), 5% and 6% respectively of cellular material was still able to be visualised on each of the surfaces.

**Re-application of Diamond Dye^TM^ to enhance visualisation for targeted sampling specific to each of the different tested surfaces**

Glass demonstrated a moderate loss in visualisation at the 6- month time point with an average of 32% of cellular material visualised prior to respraying. The number of cells that could be visualised increased significantly (p < 0.05) to an average of 79% after respraying. At the 12-month time point prior to respraying an average of 26% of cellular material were visualised compared to the 49% of cellular material visualised after respraying and this was significantly increased (p < 0.05). Overall, just over half the deposited cells persisted for the period of one year as evidenced after the DD reapplication.

At six-month post deposition and prior to respraying most cells were no longer visible on plastic substrate (on average 21% of initial deposit visualised). After respraying, 82% of cells were present indicating that for this surface the majority of cells remained at this time point, but DD fluorescence was greatly diminished. Overall, at 12-months post deposit, on average, 42% of cells remained, displaying cell persistence similar to that of glass substrate. For this surface, cell number increases from before to after respraying were significant at both time points (p <0.05).

Melamine demonstrated large loss of visualisation prior to respraying with only 5% of initial deposit still remaining, however, after the re-application of DD, approximately 62% of the initially deposited cells were present. Overall, at the last time frame, approximately 47% of cells persisted on the melamine. For this surface, again cell number increases were observed at both time points when respraying was employed, but significantly so only at 6 months (p <0.05).

For aluminium, 94% of cells persisted on the surface at 6 months post deposition (determined after respraying), however, most of the fluorescence was gone by this time point (an average of 11% of cells visualised prior to respraying). From this time point and until the final assessment at 12 months, major cell losses were observed with 50% of cells seen after the last respraying, with respraying again resulting in significant increases in cell visualisation p < 0.05 but only at six months.

For the cotton substrate drastic reduction in cell numbers were observed at six months (after respraying) indicating major losses of cells within this time period. However, cell numbers remained stable after this time period, with approximately 21% of cells still being observed at 12 months (after respraying). Respraying increased the number of cells visible by 17% and 8% respectively from before to after respraying at 6- and 12-months. This increase was significant (p <0.05) for the six months’ time point.

Similarly to cotton, leather substrate resulted in greatest cell losses with 26% and 11% of initial deposits persisting at 6- and 12-months (after respraying) and respraying again significantly improved visualisation at both time points (p < 0.05).
